# The level and determinants of empathy among medical students from Arabic speaking countries: A systematic review

**DOI:** 10.3205/zma001670

**Published:** 2024-04-15

**Authors:** Soumaya Benmaarmar, Hind Bourkhime, Ibtissam El Harch, Karima El Rhazi

**Affiliations:** 1 Sidi Mohamed Ben Abdellah University, Faculty of Medicine and Pharmacy of Fez, Department of Epidemiology, Clinical Research and Community Health, Fez, Morocco; 2 Sidi Mohamed Ben Abdellah University, Faculty of Medicine and Pharmacy of Fez, Biostatistics and Informatics Unit, Department of Epidemiology, Clinical Research and Community Health, Fez, Morocco

**Keywords:** empathy, medical students, Arabic speaking countries, systematic review

## Abstract

**Aim:**

This systematic review aims to investigate the level of empathy among medical students in Arabic speaking countries and analyze its determinants.

**Methods:**

In accordance with the Preferred Reporting Items for Systematic Reviews and Meta-Analyses 2022 (PRISMA), the authors conducted a systematic research of studies investigating the level and determinants of empathy among medical students in Arabic speaking countries. The databases PubMed, Scopus, web of science and google scholar were searched.

**Results:**

Ten studies from six countries were included. Nine of which had a cross-sectional study design. Level of empathy was assessed using the Jefferson scale in seven studies and using the Interpersonal Reactivity Index in two studies. The mean of empathy scale ranges between 97.65±14.10 to 106.55±19.16 in studies used the Jefferson scale of empathy. The associated factors with empathy were gender; high levels of empathy were reported in female students. Other factors are explored in relation with empathy such as specialty preference (surgery or medicine, “people-orientated” specialties or ‘’technology-oriented specialties’’), family factors (marital status of parents, satisfactory relationship with parents, parents level of education and household income) and factors related to medical education (academic performance, year of study and type of curriculum) but the results are heterogeneous.

**Conclusion:**

This is the first systematic review, which illustrated the determinants of empathy in Arabic medical students. Our results revealed varied results on empathy determinants. Further studies may guarantee a full exploration of this ability in order to improve the doctor-patient relationship and patient management in the Arab world.

## Zusammenfassung

**Ziel:**

Diese systematische Übersichtsarbeit zielt darauf ab, das Maß an Empathie unter Medizinstudenten in arabischsprachigen Ländern zu untersuchen und seine Determinanten zu analysieren.

**Methoden:**

In Übereinstimmung mit den Preferred Reporting Items for Systematic Reviews and Meta-Analyses 2022 (PRISMA) führten die Autoren eine systematische Recherche von Studien durch, die das Ausmaß und die Determinanten von Empathie unter Medizinstudenten in arabischsprachigen Ländern untersuchten. Es wurden die Datenbanken PubMed, Scopus, Web of Science und Google Scholar durchsucht.

**Ergebnisse:**

Es wurden zehn Studien aus sechs Ländern einbezogen. Neun davon hatten ein Querschnittsstudiendesign. Der Grad der Empathie wurde in sieben Studien mit der Jefferson-Skala und in zwei Studien mit dem Interpersonal Reactivity Index gemessen. Der Mittelwert der Empathieskala lag zwischen 97,65±14,10 und 106,55±19,16 in den Studien, die die Jefferson-Skala für Empathie verwendeten. Die mit Empathie assoziierten Faktoren waren das Geschlecht; bei weiblichen Studenten wurde ein hohes Maß an Empathie festgestellt. Andere Faktoren wurden im Zusammenhang mit Empathie untersucht, z. B. die Präferenz für ein Fachgebiet (Chirurgie oder Medizin, „menschenorientierte“ Fachgebiete oder „technologieorientierte Fachgebiete“), familiäre Faktoren (Familienstand der Eltern, zufriedenstellende Beziehung zu den Eltern, Bildungsniveau der Eltern und Haushaltseinkommen) und Faktoren im Zusammenhang mit der medizinischen Ausbildung (akademische Leistung, Studienjahr und Art des Lehrplans), aber die Ergebnisse sind uneinheitlich.

**Schlussfolgerung:**

Dies ist die erste systematische Übersichtsarbeit, die die Determinanten von Empathie bei arabischen Medizinstudenten illustriert. Unsere Ergebnisse zeigten unterschiedliche Ergebnisse zu den Determinanten der Empathie. Weitere Studien könnten eine umfassende Erforschung dieser Fähigkeit gewährleisten, um die Arzt-Patienten-Beziehung und das Patientenmanagement in der arabischen Welt zu verbessern.

## 1. Introduction

Empathy is a fundamental aspect of the communication between healthcare professionals and patients in the medical domain. Hojat et al define empathy in patient-care situations as “a cognitive attribute that involves an ability to understand the patient’s inner experiences and perspective and a capability to communicate this understanding” [1]. Indeed, the role of empathy in the doctor-patient relationship can be seen at multiple levels. In terms of biology, in diabetics and patients with dyslipidemia, empathy decreases hemoglobin A1c and low-density lipoprotein cholesterol [2]. Derksen et al. [3] have demonstrated that physician empathy is correlated with patient satisfaction, lower patients’ anxiety, distress and better clinical outcomes. Empathy decreases also the risk of burn out among healthcare professionals [4]. 

The American Medical Education Association lists empathy as one of the essential learning objectives for medical student education [5]. It is also one of the core qualities of communication mentioned in the Canadian Can MED framework for physicians ([6], p.17). 

Following these recommendations, and considering the primordial role of empathy, many studies have been conducted to evaluate the level of empathy, its evolution during the years of study and its determinants in medical students [7], [8], [9]. Results of these studies demonstrated that gender, specialty preference, socio- demographic and psychological factors can influence on empathy among students in health field. Empathy has also been found to be influenced by culture. In the systematic review by Anderson et al. [7], medical students from non-Western countries reported lower empathy scores than students from Western countries. In the study conducted by Afifi et al. [10], Arab students showed low levels of empathy compared to non-Arab students. For this reason, understanding data of this competence and its associated factors in the Arab region appears to be important to improve medical education in these countries. Thus, this systematic review aimed to explore the level of empathy and its associated factors in medical students from Arab countries. 

## 2. Methods

### 2.1. Search strategy

The review was conducted according to the PRISMA guidelines [11]. We conducted a systematic research in March 2022. Three database were searched: PubMed (MEDLINE), Scopus and web of science. The following search words were used: (empathy) AND (medical student) AND (Arab OR Algeria OR Bahrain OR Comoros OR Djibouti OR Egypt OR Iraq OR Jordan OR Kuwait OR Lebanon OR Libya OR Mauritania OR Morocco OR Oman OR Palestine OR Qatar OR Saudi Arabia OR Somalia OR Sudan OR Syria OR Tunisia OR the United Arab Emirates OR Yemen). The countries mentioned above are the countries of the Arab League. 

 In addition, a google scholar search was conducted and reference lists of eligible articles were screened for further relevant studies. 

### 2.2. Inclusion and exclusion criteria

#### Inclusion criteria

The studies that were included in this review were quantitative, original, measured level of empathy; whatever the scale of measurement used; and investigated its associated factors in medical students living in Arabic countries. No restriction was imposed concerning language and the year of publication.

#### Exclusion criteria

We excluded studies investigating the determinants of empathy in nursing students, dental students, pharmacy students or health personnel and grey literature documents (theses, conference abstracts...), qualitative and validation studies.

### 2.3. Data extraction

Studies were imported into EXCEL version 2013. After de-duplication, two reviewers independently assessed the titles and the abstracts of the articles to determine whether inclusion criteria were met. Subsequent full-text review resulted in the exclusion of additional articles. Any disagreement that arose was resolved through discussion by the two reviewers until a consensus was reached. The eligible studies were then examined by the two reviewers and relevant information of the studies was captured. This included author, study design, publication year, location, sample size, scale used to measure empathy level, the level of empathy and its associated factors. 

### 2.4. Quality assessment

The authors assessed the risk of bias (ROB) using the strengthening of observational studies in Epidemiology (STROBE) checklist [12]. It is a checklist composed of 22 items that evaluate the different types of bias. The items were grouped into 8 quality assessment criteria: sample size, sampling methodology, responses rate, outcome measures, statistical analyses, study limitation, ethical consideration and control for confounding as shown in attachment 1 , table S1. The scores assigned to each study reviewed ranged from 0 to 8 points (0 if none of the criteria were met and 8 points if all criteria were met). The sum of the assigned points represented the overall quality score of a study. Studies were classified as low quality (score ≤3); medium quality (4-6); and high quality (≥7) [13].

## 3. Results

### 3.1. Included studies

**Figure 1:**
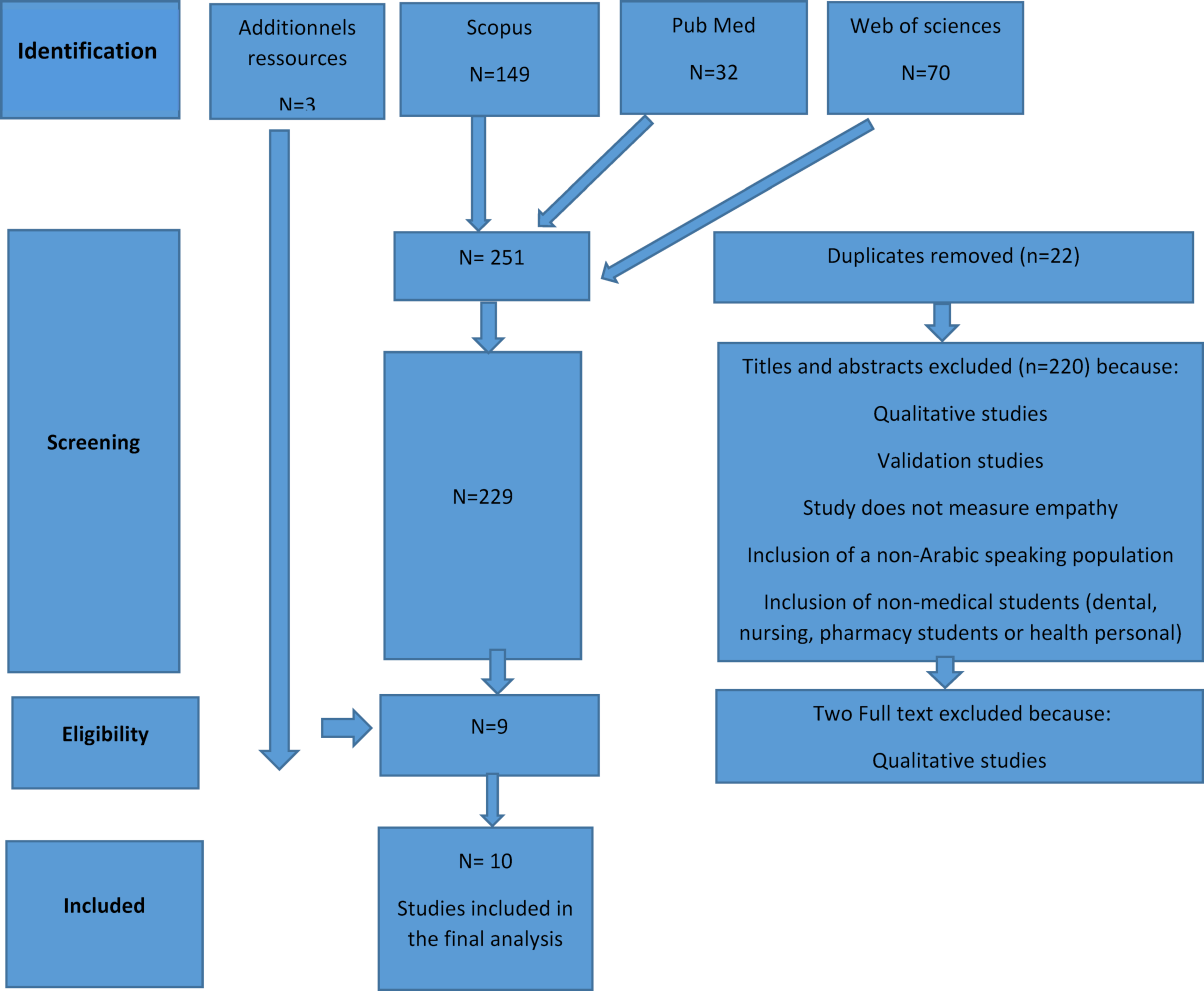
Diagram showing the search process used to identify articles included in this systematic review in accordance of PRISMA guidelines

As shown in figure 1 (Fig. 1), the search strategy resulted in 251 articles retrieved from databases, from this, 22 duplicates were found, resulting in a total of 229 articles. Following screening of the titles and abstracts, 220 articles were excluded because: they were qualitative, did not include medical students, validation studies or concerned non-Arab medical students. After full-text screening, two qualitative studies was excluded and seven articles met the inclusion criteria were included. The search in google scholar and the references of the studies identified three additional studies. A total of ten articles were analyzed.

### 3.2. Study characteristics

#### Study design and sample sizes

Of the 10 studies included in the review, 9 studies were cross-sectional [10], [14], [15], [16], [17], [18], [19], [20], [21] and 1 study was longitudinal [22]. Sample sizes of the cross-sectional studies varied from 40 [21] to 1110 Participants [14]. Concerning longitudinal study, sample size was not specified [22] (see attachment 2 ).

#### Scales 

Level of empathy was assessed using the Jefferson scale in seven studies [14], [15], [16], [17], [18], [19], [20], and the Interpersonal Reactivity Index (IRI) in two others [10], [21]. In study of ARAIN et al, the scale of measure was not specified [20]. 

#### Country

The studies were conducted in six different Arabic countries: Saudi Arabic [14], [15], [19], [20], Lebanon [22], Iraq [17], Kuwait [16], Morocco [18] and the United Arab Emirates [10], [21].

#### Quality assessment and risk of bias in the included studies

The scale to evaluate the quality of included studies showed that eight studies were of medium quality, while two studies were of a low quality. The details of quality assessment are provided in attachment 1 , table S2.

#### Level of empathy

In studies that measured empathy using the Jefferson scale of empathy, the average score ranged from 97.65±14.10 [18] to 106.55±19.16 [14]. The mean of empathy score in the study using IRI, was 64.7±12.3 in study of AFIFI et al. [10]. It was 54.9±5.6 in female and 51.8±7.1 in male in study of Hashim et al. [21].

#### Gender

 Of the ten studies, eight explored the association between gender and level of empathy [14], [15], [16], [17], [18], [19], [20], [21]. Female students had significantly higher empathy scores than male students in some studies [16], [17], [18], [19], except in study of Arain et al. where the inverse was observed [20]. In other studies, the level of empathy was higher in female students but no significant differences were detected [14], [15], [21]. 

#### Evolution of the empathy score across the educational levels 

Five studies have assessed the evolution of students' empathy levels over the academic years [14], [16], [17], [19], [22]. Two studies reported that scores of empathy decreased as educational level increased [19], [22]. In the Iraqi study [17], the decline in the score of empathy was found until the fourth year. One study reported a higher level of empathy among medical students at an advanced year of medical school [14]. In the study conducted in Kuwait [16], the level of empathy increased until the fourth year and then decreased. 

#### Specialty preferences

Four cross-sectional studies [14], [15], [16], [17] investigated a possible relation between empathy scores and specialty preferences of the students. One study detected higher levels of empathy among students who preferred a “people-orientated” specialties than those who selected technology-oriented specialties [17]. Another showed higher levels among student who preferred surgery than those who preferred medicine [15]. In the remaining two studies [14], [16], no statistically significant differences in the empathy level among the students with different preferred specialty was found. 

#### Family factors

Studies of Hassan et al. [16] and Ayuob et al. [14] explored the relationship between the level of empathy and family factors of students such as: parents education, family income, marital status of parents, satisfactory relationship with parents and having a sick relative. In both studies, empathy was associated with satisfactory relation with parents in family. In study of Hassan et al. [16], high family income and high educational level of mother and having a sick relative increase the level of empathy. In Saudi Arabian study [14], stable marital status of parent during medical education, was associated with high level of empathy. 

#### Psychological factors: stress and personality trait

The association between stress and empathy was studied only in the study of Hassan et al. [16] and showed a positive correlation. The same study [16] has investigated the relationship between five different aspects of personality (aggression and hostility, impulsive sensation seeking, neuroticism and anxiety, activity, and sociability) and level of empathy but no significant association was found.

#### Academic factors

##### Academic achievement

Three studies investigated the association between academic achievement and empathy [15], [16], [19]. Two studies [15], [19] found that high levels of empathy was associated with good academic performance and one study [16] did not show a significant association. 

##### Internships circumstances

The Moroccan study [18] has explored the impact of internships circumstances (transportation time, the distance between the housing and the hospital) on empathy but no significant association was found.

#### Curriculum

In the Lebanese study [22], the authors investigated the impact of the new medical curriculum that aimed to enhance the student’s empathy, compassion, and advocacy by the introduction of the physicians patients and society courses, the social medicine and global health course and the learning communities and clinical skills courses. Higher empathy scores were observed for the students who followed the new curriculum than those who followed the traditional based only on fundamental sciences.

#### Language of communication courses

Study of Hashim et al. [21] showed that learning communication courses in a language, which is not the students’ native language, was associated of low students’ level of empathy. 

## 4. Discussion

This systematic review aimed to investigate the level of empathy and its determinants among Arabic medical students. The results of the included studies demonstrated a various levels of empathy. Exploration of related factors showed high levels of empathy in female students. It showed also that other factors (socio-demographic, cultural, academic, and psychological) influence the level of empathy but the results are various. 

The level of empathy ranges between 97.65±14.10 to 106.55±19.16 in studies used Jefferson scale. These scores were lower than those in USA [23], United Kingdom [24], Argentina [25], Spain [26], Peru [27] and Brazil [28] and higher than those found in Portugal [29], Pakistan [30], India [31] and Iran [32]. This difference may be due to cultural, psychological and social differences between countries [33]. The Saudian students have obtained the highest empathy scores [14], [15], [19]. 

The evolution of the level of empathy over the years of medical education is very heterogeneous in this review. Some studies showed that the level increased over the years and others showed a low level of empathy in last year of study. These results are consistent with results of a recent systematic review that not specific to Arab context [7]. The reasons for the decline in empathy over the medical educational levels are explained in the review by Neumann et al. [8]; who identified distress (burnout, reduced quality of life, depression, stress) as the main factor influencing empathy in medical students. Aspects of the hidden curriculum such as maltreatment by superiors, lack of social support from family, vulnerability of students to illness and death may contribute to the decline in empathy. Furthermore, inadequate learning environments and lack of appropriate role models are also cited as possible reasons for the decline in empathy. However, the increase in empathy levels during medical school years may reflect the influence of the medical training program based on courses to develop empathy and the doctor-patient relationship, more exposure to clinical training, and more interaction with patients [8], [34].

Most studies found a tendency towards higher levels of empathy among female students as compared to male students. This result was in line with literature data [7]. The high level of empathy in women is related to the fact that women are more responsive to emotional cues, more understanding of feelings, and more attentive to patients than men, which leads to a better empathic relationship [35]. Biological and genetic factors may also explain this difference [36].

In this review, the impact of psychological factors on students' empathy levels is poorly studied. Only one study [16] has explored the impact of stress and found its positive correlation with the empathy levels. However, previous studies [37] reported that stress is major factors in empathy decline among medical students. The same study [16] did not show an association between personality traits and the level of empathy among students, as opposed to the data in the literature which confirmed that empathy is related to personality [26], [37], [38], [39], [40].

The impact of student’s family factors on their empathy skill is rarely studied in selected studies and even the literature and the results are controversal. High schooling of mother was associated with higher level of empathy in study of Hassan et al. [16]. In contrast, a Brazilian study [41] demonstrated that students whose parents did not have a higher education degree showed higher empathy levels than those who had at least one parent with a high education degree. In this review, students who are satisfied with their relationship with their parents had higher level of empathy, the same observation was found by Hojat et al. [42]. One study in the current review [14] demonstrated that an experience of a patient in the family was significantly associated with high empathy scores. On the other hand, other studies [41], [43] did not show any association between empathy score and family history of chronic or severe disease. 

Besides the factors described above, the education environment and curricular model practiced in the different Medicine Universities can also influence on increasing or decreasing the level of empathy. In this review, the Lebanese study [22] revealed the influence of teaching scheme on the levels of medical student’s empathy. Similar findings were noted in a various studies [29], [44], demonstrating that the curriculum based on courses aimed to enhance communication skills and the doctor-patient relationship may increase empathy skill. The Lebanese study [22] stands out in quality because it is a longitudinal study with multiple cohorts that examines not only empathy but also the learning environment and aspects of the hidden curriculum. This could be highlighted as an exemplary study.

In the cohort of Hashim et al. [21], the language of learning communication skills in a language other than the native language had a negative influence on the degree of empathy. Similar results have been found in other studies [45], [46]. In agreement with results from previous research [34], [47], two studies demonstrated that higher academic achievement were associated with higher level of empathy. 

This review has some limitations: The first one is related to some methodological difficulties. Some studies had small sample sizes [15], [21] and all studies were conducted in a single university, making the results of these studies less generalizable and affecting the external validity. Only one study [14] of the ten was used the JSPE scale translated and validated in Arabic and in the survey of Arain et al. [20] there is a lack of information about the scale used for measure of empathy, its scoring and its psychometric properties, which can produce an information bias. The majority of studies explored a minimal number of factors except for the study conducted in Kuwait [16], which studied the different factors: socio-demographic, academic, family and psychological. Lastly, it is possible that some relevant studies were unintentionally excluded from the analysis even though the research was conducted in several electronic databases. 

In conclusion, this is the first review exploring empathy in Arab medical students. However, it includes only ten studies, in all Arab countries, with some methodological limitations. Given the current state of research, it is not possible to fully determine the factors associated with the level of empathy among Arabic-speaking medical students. This highlights the need for more effective studies that take into account cultural, sociodemographic, psychological, familial, and academic factors. Longitudinal studies are also recommended to investigate the evolution of empathy over the years of study. As noted above, studies that mainly concern the level of empathy and its determinants are based on empathy scales administered mostly in English. However, validation studies of these scales are needed to ensure the validity and pertinence of the results. We also recommend further systematic reviews including studies of empathy in healthcare professionals and other students in the medical field. These studies will improve knowledge about the level of empathy and its determinants, which will positively influence the learning of this skill in Arab medical schools. 

## Funding

This work was supported by the Sidi Mohamed Ben Abdellah University, Faculty of Medicine and Pharmacy of Fes and Hassan II University Hospital of Fez.

## Ethical approval

Due to the nature of the study, no ethical approval was needed.

## Competing interests

The authors declare that they have no competing interests. 

## Supplementary Material

Supplementary tables

Characteristics of included studies
