# Supplementary material for: The level and determinants of empathy among medical students from Arabic speaking countries: A systematic review
Source: GMS J Med Educ. 2024 Apr 15;41(2):Doc15. doi: 10.3205/zma001670 (PMC11106575; doi:10.3205/zma001670)
Supplement: Supplementary tables [file JME-41-15-s-001.pdf]

Attachment 1: Supplementary tables

Supplementary table 1: Assessment tools used for the quality appraisal of the included studies, based on STROBE checklist (8 quality assessment criteria)

**Sample size**	**Sampling methodology (Represen-tativeness)**	**Responses rate**	**Outcome measure**	**Statistical analysis**	**Control of confounding**	**Study limitations**	**Ethical considerations**
[1 point] Study reported sample size calculation	[1 point] Random selection from representative list of the target population	[1 point] High response rate (>70%)	[1 point] Valid acceptable scale of measure empathy	[1 point] Statistical techniques and reasons for choosing techniques are fully explained	[1 point] Well adjusted (Individual, household and community level factors). Adjusted and unadjusted Odd Ratio (OR) or Relative Ratio (RR), confidence interval and *p*-values were well presented	[1 point] Stated study limitations	[1 point] Studies with ethical consideration
[0 points] No sample size calculation	[0 points] Nonrandom selection but from sampling frame that represent the community	[0 points] Moderate/low response rate (<70%)	[0 points] No valid scale of measure empathy	[0 points] Statistical techniques are not explained	[0 points] Minimal adjusted for some or none of the confounders	[0 points] No stated study limitations	[0 points] Studies with no ethical consideration

Supplementary table 2: Quality assessment of included studies

**Author, Year Country**	**Sample size**	**Sampling methodology (Represen-tativeness)**	**Responses rate**	**Outcome measure**	**Statistical analysis**	**Confounding**	**Study limitations**	**Ethical consider-ations**	**Quality assessement**
Hamed et al. 2015	1	0	-	1	1	0	1	1	5
Hasan et al. 2013	0	0	0	1	1	0	1	1	4
Iqbal et al. 2022	1	0	0	1	1	0	1	1	5
Ayuob et al. 2016	0	0	0	1	1	0	1	1	4
ARAIN et al. 2019	0	0	-	0	1	0	1	1	3
Jaafari et al. 2018	0	0	-	1	1	0	0	1	3
Raof. Yassin et al. 2016	0	0	1	1	1	0	1	1	5
Zgheib et al. 2020	0	0	-	1	1	0	1	1	4
Hashim et al. 2013	0	0	1	1	1	0	1	1	5
Afifi 2018	0	0	-	1	1	1	1	1	5
