# Supplementary material for: The level and determinants of empathy among medical students from Arabic speaking countries: A systematic review
Source: GMS J Med Educ. 2024 Apr 15;41(2):Doc15. doi: 10.3205/zma001670 (PMC11106575; doi:10.3205/zma001670)
Supplement: Characteristics of included studies [file JME-41-15-s-002.pdf]

Attachment 2: Characteristics of included studies

Study number	Title	Author, published year	Country	Objective	Study design	Sample size (response rate)	Scale	Empathy level	Determinants
1	Is the Empathy Level of Medical Students at KAU Changeable along their Study Years? What is behind this Change?	Ayuob et al. 2016	Saudi Arabia	To determine the level of empathy among medical students in different study years in the Faculty of Medicine (FOM) at King Abdulaziz University (KAU) and to explore in depth the factors that affect it.	Cross-sectional	1110 (58.3%)	JSPE-S (Arabic version)	106.55±19.16	**- year of study:** Level of empathy increases along with the study year<br>**- family factors:** stable marital status of parents, satisfactory relationship with mother and father, having a sick relative, were associated with higher level of empathy
2	Personal, Cultural and Academic Factors Affecting Empathy Score in Third Year Medical Students	Hamed et al. 2015	Saudi Arabia	Evaluating the empathy scores in third year undergraduate medical Students in King Abdulaziz University and explore the factors which might have an effect on them and how might these factors affect them.	Cross-sectional	163	JSPE-S	M: 100.1±17.3<br>F: 103.7±12.2	- **specialty preference:** Higher scores were observed among students who selected "surgery" rather than “medicine” as their future specialties.<br>-**Grade point average:** Students who has a higher GPA showed significantly higher scores in level of empathy

Study number	Title	Author, published year	Country	Objective	Study design	Sample size (response rate)	Scale	Empathy level	Determinants
3	Level of Empathy among Medical Students in Kuwait University, Kuwait	Hasan et al. 2013	Kuwait	To evaluate the level of empathy among medical students in Kuwait University Medical School and its association with socio-demographic factors, stress levels and personality.	Cross-sectional	264 (56%)	JSPE-S (English version)	104.6±16.3	- **gender:** higher level of empathy among female students<br>- **year of study:** the level of empathy increased until the 4th year and then decreased<br>- **family factors:** higher mother's level of education, high household income, satisfactory relationship with the mother were associated with higher level of empathy<br>- **stress levels:** Stress scores were positively associated with empathy
4	Measuring Empathy Levels among Kurdish Medical Students in Erbil City, Iraq	Raof. Yassin et al. 2016	Iraq	To evaluate the empathy levels of students in the College of Medicine at Hawler Medical University (HMU) in Erbil city, Iraq.	Cross-sectional	927 (93.7%)	JSPE-S (English version)	101.9±19.2	**- gender:** higher level of empathy among female students<br>- **specialty preference:** students who chose people-oriented specialties had higher mean empathy scores, whereas those who selected technology-oriented specialties had lower scores<br>- **year of study:** Mean empathy scores decreased as academic years increased until the fourth year

Study number	Title	Author, published year	Country	Objective	Study design	Sample size (response rate)	Scale	Empathy level	Determinants
5	Cross sectional study on lifelong learning's determinants among medical students in RAK Medical & Health Sciences University, UAE	Afifi 2018	The United Arab Emirates	To find the association of Empathy, Self-Efficacy, and/or hope with readiness for lifelong learning among medical students.	Cross-sectional	n=221<br>Arab<br>n= 104	IRI	Arab:<br>64.7±12.3	The associated factors are studied in the overall population (Arab and non-Arab students) and not studied for the Arab population alone
6	Effect of Targeted Curricular Reform on the Learning Environment, Student Empathy, and Hidden Curriculum in a Medical School: A 7-Year Longitudinal Study	Zgheib et al. 2020	Lebanon	To assess the effectiveness of a new curriculum in empathy over a period of 7 years.	Longitu-dinal	-	JSPE-S	Not specified	**- curriculum:** the new curriculum increase the level of empathy<br>**- year of study:** Decline in the level of empathy by increase in number of educational years.
7	Measuring empathy in medical students: A cross-sectional study	Iqbal et al. 2022	Saudi Arabia	To measure the empathy levels of undergraduate medical students, and to 18 explore whether the empathy level has any significant association with gender, 19 academic year and academic achievement.	Cross-sectional study	391(43,8)	JSPE-S (English version)	105.18±12.51	- **gender:** Female students had significantly higher empathy scores than males<br>- **GPA (grade point average):** Students with a higher GPA had higher levels of empathy level than students with a lower GPA<br>- **year of study:** decrease of levels of empathy by increase in number of educational years

Study number	Title	Author, published year	Country	Objective	Study design	Sample size (response rate)	Scale	Empathy level	Determinants
8	Medical Students Learning Communication Skills in a Second Language: Empathy and expectations	Hashim et al. 2013	The United Arab Emirates	To identify whether Arabic-speaking medical students experienced difficulty with the different components of the CS training that were conducted in English.	Cross-sectional*	40 (89%)	IRI (English version)	F : 54.9±5.6<br>H: 51.8±7.1	**Learning communication skills in a second language:** was associated with low empathy scores
9	Level of Patient Empathy among medical students of Saudi Medical College: A Cross-sectional Survey	ARAIN et al. 2019	Saudi Arabia	1. To measure the mean level of empathy among medical students.<br>2. To compare the level of empathy among various academic years and among gender.	Cross-sectional	545	Not specified (English version)	65.21±7.24	**gender**: Mean score of empathy was found to be better among male students compared to female students
10	A measurement of empathy among Moroccan medical students	Jaafari et al. 2018	Morocco	To determine the impact of gender and year of study on empathy in medical students.	Cross-sectional	632	JSPE (version French)	97.65±4.10	**gender:** Female students had significantly higher empathy scores than males

JSPE: Jefferson Scale of Physicians Empathy, JSPE-S: Jefferson Scale of Physicians Empathy (student version), IRI: Interpersonal Reactivity Index.
* This is a longitudinal study, but empathy is only measured once. So, in terms of empathy, the study was considered cross-sectional.
